# Age-dependent impairment of cardiac function and physical performance in male mice with diet-induced obesity

**DOI:** 10.1007/s11357-025-01775-7

**Published:** 2025-07-10

**Authors:** Patricia Baumgarten, Justus Kamp, Niklas Hegemann, Stefanie Deubel, Nikolaus Berndt, Jana Grune, Wolfgang M. Kuebler, Christopher L. Axelrod, John P. Kirwan, Annika Höhn, Sophie Heider, Christiane Ott, Tilman Grune

**Affiliations:** 1 Department of Molecular Toxicology, German Institute of Human Nutrition Potsdam-Rehbruecke, Nuthetal, Arthur-Scheunert-Allee 114-116, 14585 Germany; 2 German Center for Cardiovascular Research (DZHK), Berlin, Partner Site Berlin, Potsdamer Straße 58, Germany; 3 Department of Cardiothoracic and Vascular Surgery, Deutsches Herzzentrum Der Charité (DHZC), Berlin, Augustenburger Platz 1, 13353 Germany; 4 Charité – Universitätsmedizin Berlin, corporate member of Freie Universität Berlin and Humboldt-Universität Zu Berlin, Berlin, Charitéplatz 1, 10117 Germany; 5 Deutsches Herzzentrum Der Charité (DHZC), Institute of Computer-Assisted Cardiovascular Medicine, Berlin, Augustenburger Platz 1, 13353 Germany; 6 German Center for Lung Research (DZL)M, Associated Partner Site Berlin, Berlin, Germany; 7 Keenan Research Centre, St. Michael´S Hospital, Toronto, ON Canada; 8 Departments of Physiology and Surgery, University of Toronto, Toronto, ON Canada; 9 Integrated Physiology and Molecular Medicine, Pennington Biomedical Research Center, Baton Rouge, LA USA; 10 German Center for Diabetes Research (DZD) Ingoldstadter Landstraße 1, Munich-Neuherberg, 85764 Germany; 11 Institute of Nutritional Science, University of Potsdam, Potsdam, 14469 Germany; 12 Department of Physiological Chemistry, Faculty of Chemistry, University of Vienna, Vienna, 1090 Austria

**Keywords:** Aging, Physical performance, Diet-induced obesity, Heart failure, Heart function

## Abstract

Aging in the context of obesity exacerbates the risk of morbidity and mortality related to cardiovascular disease. However, the maladaptive responses in the heart that arise from prolonged obesity and the specific influence of biological age remain somewhat elusive. This study investigated the effects of diet-induced obesity (DIO) and aging on physical performance and cardiovascular function in mice. 22- and 76-week-old male C57BL/6J mice were randomized to 8 weeks of chow or high-fat diet. Body weight was assessed weekly. Body composition was measured at the beginning and the end of the diet treatment. Muscular and cardiac function were evaluated at the end intervention. Aged mice with DIO exhibited faster and greater body weight gain and fat mass accumulation, reduced running distance, and lower aerobic capacity. Aged HFD mice also exhibited increased cardiac lipid accumulation and cardiomyocyte hypertrophy, with no major morphological changes observed in skeletal muscle. Proteomic analysis revealed differential expression of heart proteins associated with metabolic function in young mice, which was not observed in aged mice with DIO. Subsequently, aged mice with DIO developed overt heart failure with reduced ejection fraction, while cardiac function was unaffected by DIO in young mice. In conclusion, young mice with DIO were protected against diet-induced cardiac dysfunction, whereas DIO in aged mice led to heart failure and impaired physical performance. The protective effects observed in younger mice appear to be explained by proteomic-level remodeling of the heart oriented to sustain cardiac function.

## Introduction

Obesity, defined by a body mass index (BMI) of ≥ 30 kg/m^2^, and its related, imposes significant economic and personal burden [1–3]. To this end, obesity is independently associated with the development of cardiovascular diseases (CVDs), diabetes, chronic kidney disease, liver disease, and cancer [2]. Among these, heart disease remains the primary cause of death in individuals with obesity [4]. Furthermore, obesity and its comorbidities are linked to decreased life expectancy [4, 5]. It has been postulated that obesity accelerates the aging process and may directly lower lifespan [6]. Specifically, individuals with a BMI ≥ 40 kg/m^2^ face a reduction in life expectancy of 6 to 13 years due to the cumulative risk of comorbidities [4, 7]. While the effects of obesity and aging are well studied on their own, it is less clear how aging influences the body’s response to obesity [8].

With the increasing prevalence of obesity and an aging global population, understanding the pathological mechanisms underlying CVDs, particularly in the context of aging and obesity, has become essential for addressing this growing public health challenge [1, 8]. It is well-established that cardiac metabolic alterations occur in patients with heart failure [9], alongside morphological and cellular alterations [10]. In addition to cardiac changes, heart failure induces alterations in skeletal muscle, contributing to physical limitations and immobility [11].

The aim of this study was to investigate the impact of obesity in the context of aging on metabolic alterations, physical performance, and cardiac function. To explore the metabolic response to HFD feeding in both young and aged mice, we employed a variety of methods, including assessments of overall body composition, physical performance, muscle morphology, and a detailed focus on the morphological, metabolic, and functional changes in the heart. We observed that aged mice with diet-induced obesity (DIO) were more susceptible to the accrual of fat mass and cardiac lipid infiltration compared to young mice with DIO. This phenotype was explained, in part, by proteomic-level adaptations associated with metabolic function in young mice with DIO, which was absent in aged mice with DIO. Finally, we show that aged mice with DIO develop impaired heart function and ultimately decreased physical performance. Taken together, these observations support the notion that obesity accelerates metabolic aging and contributes to cardiovascular health decline in mice.

## Materials and methods

### Mouse study

All animal procedures were performed in accordance with the Guide for the Care and Use of Laboratory Animals (Institute of Laboratory Animal Resources, 7th edition, 1996) and the European legislation for animal welfare (Directive 2010/63/EU). All procedures were approved by the local authorities (Approval number: 2347–46-2019) and adhered to institutional guidelines.

Male C57Bl/6J mice (bred in-house at the German Institute of Human Nutrition, Potsdam-Rehbruecke, Germany) were housed in a controlled environment (20 ± 2 °C, 12/12 h light/dark cycle) with ad libitum access to food and water. After weaning, the mice were maintained on a rodent chow diet (V1534-300, Ssniff, Soest, Germany). Mice were randomly divided into two different age groups, which were further randomly assigned to one of two diet groups. The intervention phase lasted 8 weeks, during which the mice received either the chow diet as a control or a high-fat diet (HFD; Bio-Serve, Flemington, US), composed of 60% energy from fat. At study end, the young mice were 22 weeks (young), and the old mice were 76 weeks of age (aged). While intervention lengths are varying in DIO mice studies, our study employed an 8-week HFD intervention to focus on the early metabolic adaptations to obesity before the onset of more severe comorbidities like type 2 diabetes. This time frame allowed us to explore metabolic shifts without the confounding effects of advanced metabolic syndrome. During the intervention phase, mice underwent weekly body weight and blood glucose measurements.

### Blood and organ collection

After sacrifice by acute isoflurane exposure followed by cervical dislocation, blood was collected using an EDTA rinsed syringe from *vena cava inferior,* centrifuged at 4 °C for 5 min at 14,000 rpm to separate plasma from remaining components. Heart and quadriceps tissue were collected, snap-frozen in liquid nitrogen, and stored at − 80 °C for further analysis.

### Body composition analysis

Body composition measurements of fat mass and lean mass were performed using nuclear magnetic resonance (NMR) spectrometry with an EchoMRI analyzer (Houston, TX, USA)[12]. Mice remain fully conscious, and each mouse is placed briefly in an open cylindrical chamber, and the scan itself takes only 30–60 s, thereby minimizing handling stress. Measurements were obtained twice for every animal: once immediately before the intervention began and again 1 week prior to sacrifice. For interpretation, raw values were normalized to the body weight by dividing the recorded fat or lean mass by the concurrent body weight. In addition, the change over time (Δ) was calculated for each animal by subtracting the value of the first measurement from the second.

Additionally, the epididymal fat pad was removed and weighed to calculate the adipose index in percentage to the total body weight as described earlier [13].

$$Adipose index \left[\%\right]= \frac{Epididymal fat pad [g]}{Body weight [g]}*100$$

### Physical performance

A metabolic treadmill was used to assess physical performance and measurements by indirect calorimetry [14]. It was a non-voluntary activity test using electrical shocks to motivate the mice to run. The metabolic treadmill (Columbus Instruments, Columbus, OH, USA) assessed oxygen (O_2_) and carbon dioxide (CO_2_) levels, allowing for the calculation of volume of CO_2_ (VCO_2_) or O_2_ (VO_2_), as well as the respiratory exchange ratio (RER). On the experiment day, each mouse performed the same protocol until the stop criteria were reached. Stop criteria were defined as follows: 3 s on the electrical grid, a respiratory exchange ratio (RER) above 1, or a maximum running time of 1 h. The protocol was set as follows: 5-min acclimatization phase in the chamber without running, followed by an increase in running speed every 2.5 min, starting from 7 m/min, then increasing to 10 m/min, 15 m/min, and finally reaching 20 m/min. During the run, the treadmill was set at a 10° incline.

### Grip strength

Muscle strength was measured before and after the intervention phase using a Grip Strength Meter (Bioseb, Vitrolles Cedex, France). The maximum grip strength (gf) was quantified as the average of three force measurements.

### Histological characterization

To evaluate changes in morphology, both heart and quadriceps tissues were subjected to histological analysis. After tissue collection, samples were placed in tissue embedding cassettes and fixed in 4% formalin for at least 24 h at RT. Following fixation, tissues were then embedded in paraffin, and 2 µm and 4 µm cross-sections were prepared. For Hematoxylin & eosin (H&E) staining of quadriceps, deparaffinized slides were incubated with Hematoxylin dye (Roth, T 865.3) for 3 min and 1 min with Eosin solution. To measure the cross-sectional area of fibers in quadriceps tissue, at least 100 fibers from two different areas of the H&E-stained slides per sample were analyzed using ImageJ. For picosirius red staining of the heart, deparaffinized slides were incubated with picosirius red solution (Direct Red 80, Sigma-Aldrich, 365548) for 1 h at RT. Total collagen levels were calculated by dividing the red-stained collagen area by the total heart area. To evaluate the size of cardiomyocytes and assess cell hypertrophy, Wheat Germ Agglutinin (WGA-Alexa Fluor 488, Invitrogen, USA) staining was used, following the protocol described by Bensley et al. (2016) [15]. WGA was applied at a concentration of 10 µg/ml for 15 min in the dark, followed by washing steps and mounting with DAPI (Roth, HP20.1). After imaging, the size of at least 100 cardiomyocytes from two different areas per slide was evaluated, and the average cross-sectional area of all cells is reported in µm^2^. After staining, slides were imaged using the Axio Scan 7 (Zeiss, Oberkochen, Germany), and analysis was performed using Zeiss ZEN 3.0 Blue Edition and ImageJ.

### Proteomic analysis

Approximately 5 mg of snap-frozen mouse heart tissue was lysed with 75 µl of buffer (1% SDS, 100 mM ABC, 1.25 × PIC) and processed with sonication (Covaris LE220Rsc). After centrifugation, 50 µl of lysate was transferred to a 96-well plate and automated on a Biomek i7 workstation (Müller et al., 2020). Reduction/alkylation (4% SDS, 40 mM TCEP, 160 mM CAA, 200 mM ABC) was performed at 95 °C for 5 min. Proteins (~ 50 µg) were bound to 500 µg paramagnetic beads in 50% ACN, washed, and reconstituted in 35 µl of 100 mM ABC. Digestion was carried out with Trypsin/LysC (37 °C), stopped with 5% formic acid, and prepared for Liquid Chromatography Tandem Mass Spectrometry (LC–MS/MS). Peptides (~ 1 µg) were separated on a trap (PepMap C18) and analytical (Acclaim PepMap C18) column using an Ultimate 3000 HPLC coupled to a Q-Exactive Plus mass spectrometer in DIA mode. Raw data were analyzed with DIA-NN 1.8 (PMID: 31,768,060) using a mouse UniProt spectral library, refined automatically (0.01 global q-value) and filtered at 1% peptide-level FDR (Messner et al., 2020; UniProt Consortium, 2019).

Principal component analysis (PCA) was performed using MATLAB’s “pca” function to identify components capturing the most significant variance in the proteomic dataset. Statistical analysis included two-sample *t*-tests with Benjamini–Hochberg correction, visualized in a volcano plot showing log2 fold changes and adjusted p-values (negative values for downregulated, positive for upregulated proteins). Analysis was conducted with MATLAB 2023b (The MathWorks) and the bioinformatics toolbox. Pathway analysis was performed via ShinyGO (v0.81) for KEGG pathway enrichment. Heatmaps of selected targets were generated in GraphPad Prism. Raw counts were log10-transformed, *Z*-score normalized (mean-centered, divided by SD), and plotted (blue: downregulation, red: upregulation).

### Echocardiography

The methodology is described in detail by Grune et al. (2018) and Ott et al. (2021) [16, 17]. For echocardiographic analysis, mice were anesthetized using isoflurane and placed on a heated platform in a supine position. The paws were taped to prevent movement, and the hair around the measurement area was removed. Echocardiography of both ventricles was performed using a Vevo 3100 high-resolution imaging system coupled with an MX400 ultra-high-frequency linear array transducer (18–38 MHz; center transmit: 30 MHz; axial resolution: 50 μm) (both FUJIFILM VisualSonics, Toronto, Ontario, Canada). Images were captured in the parasternal long-axis view (PLAX) in both B-Mode and M-Mode. All acquired images were digitally stored in raw format (DICOM) for further offline analysis. Image analyses were performed by a trained expert in small animal echocardiography using the dedicated software package VevoLAB Version 3.1.0 (FUJIFILM VisualSonics).

### Statistical analysis

The results were statistically analyzed using GraphPad Prism Software (version 10.3.0). The results are expressed as mean ± SD. Outliers were identified using the ROUT method for nonlinear regression. Normal Gaussian distribution was assessed using the Shapiro–Wilk normality test. Data were then grouped by age and diet, and analyzed using two-way ANOVA, followed by Tukey’s multiple comparison test. Statistical significance is indicated as follows: *p-value < 0.05, **p-value < 0.01, ***p-value < 0.001, ****p-value < 0.0001. Analysis of simple linear regression was used to assess the relationship between body weight and various functional parameters, showing the 95% confidence bands of the best-fit line, as well as the *R*^2^ and the *p*-value.

## Results

### HFD-induced weight gain and fat mass accumulation are more pronounced in aged mice

**Fig. 1:**
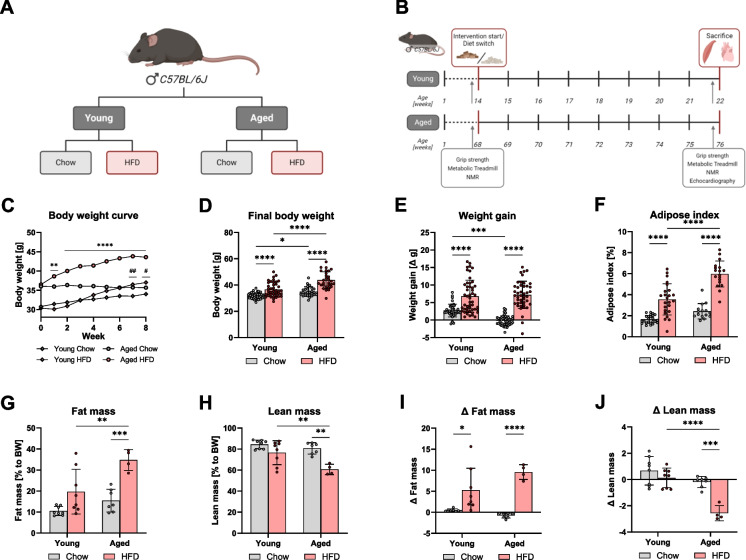
The increase in body weight was primarily due to fat mass accumulation, particularly in the aged group. Male C57Bl/6J mice were randomized into four groups: two age groups (22 weeks = young, 76 weeks = old) receiving either a chow diet or a High-Fat Diet (HFD) during an 8-week intervention period (**A**). Before and after the intervention, body composition was assessed by nuclear magnetic resonance (NMR), and physical performance was evaluated using grip strength and a metabolic treadmill test. At the end of the study, echocardiography was performed, and the mice were sacrificed. Plasma, quadriceps muscle, and heart tissue were collected (**B**). Body weight was monitored through weekly measurements. Statistical significance was tested using two-way ANOVA; # indicates statistical significance between young chow vs. HFD, while * indicates significance between old chow vs. HFD (**C**). Final body weight before sacrifice (**D**) and weight gain from the start to the end of the intervention (**E**) are shown. The adipose index was calculated as follows: (epididymal fat pad weight/body weight) * 100 (**F**). NMR spectrometry data are presented as a percentage of body weight (**G, H**) or as the difference between the two measurements to illustrate changes during the intervention (**I, J**). Data are presented as mean ± SD. Statistical significance was tested using two-way ANOVA, with significance levels indicated as: *p < 0.05, **p < 0.01, ***p < 0.001, ****p < 0.0001

To investigate age-dependent metabolic responses to overfeeding, we used a DIO mouse model (Fig. 1A, B). Body weight measurements revealed that the mice of both age groups gained weight similarly in response to HFD (Fig. 1C, E). Final body weight was increased by HFD in both age groups. The body weight was higher in aged mice compared to young mice, regardless of diet (Fig. 1D). The overall weight gain from the start to the end of the intervention showed an increase in both age groups on HFD (Fig. 1C, E). Additionally, young mice on chow diet experienced weight gain that was not present in the aged mice on the chow diet (Fig. 1E). In addition to body weight, we evaluated body composition using the adipose index and NMR analysis. The adipose index increased in both age groups following HFD, with a higher adipose index in aged mice compared to young mice on HFD (Fig. 1F). Fat mass, expressed as a percentage of body weight, increased in the old HFD group but not in the young HFD group compared to chow-fed mice. Fat mass was higher in the aged HFD group compared to young mice on HFD (Fig. 1G). Lean mass decreased in aged mice on HFD compared to aged mice on chow and young mice on HFD (Fig. 1H). The delta fat mass showed an increase in fat mass in both age groups on HFD, while only aged mice on HFD exhibited a significant decrease in delta lean mass compared to chow-fed mice (Fig. 1I, J). This is also true if the absolute weights were related to the tibia length (Fig. S2). Collectively, these results confirm the DIO in mice, driven by an increase in body mass and fat accumulation, and therefore, are more inclined to develop obesity.

### Physical performance is impaired in aged mice on HFD

**Fig. 2:**
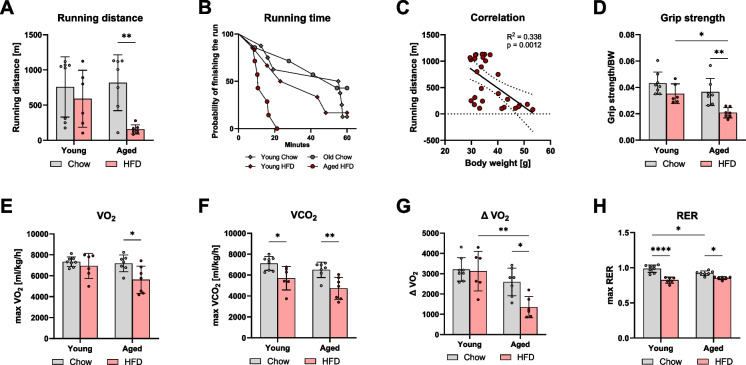
The high-fat diet feeding led to impaired physical performance in aged mice. Maximal running distance on a metabolic treadmill test (**A**). Kaplan–Meier survival analysis showing the probability of completing the run within the 60-min maximum time (**B**). Simple linear regression analysis of all groups, correlation of running distance to body weight (**C**). Grip strength normalized to body weight (**D**). Indirect calorimetry measurements during the run, including max oxygen consumption (VO2, **E**), max carbon dioxide production (VCO2, **F**), the change in oxygen consumption from the start to the end of the run (Delta VO2, **G**), and the respiratory exchange ratio (RER, VCO2/VO2) (**H**). Data are presented as mean ± SD. Statistical significance was tested using two-way ANOVA, with significance levels indicated as: *p < 0.05, **p < 0.01, ***p < 0.001, ****p < 0.0001

To assess physical performance, we initially performed a metabolic treadmill test. As shown in Fig. 2A, B, the aged HFD group exhibited a lower running distance and time compared to the aged chow-fed group, while the young mice showed no effect of HFD on running distance. Simple linear regression analysis of running distance relative to body weight revealed a significant negative correlation (*p* < 0.0012, *R*^2^ = 0.338), indicating that higher body weight was associated with shorter running distance (Fig. 2C). Another, well-accepted test for muscle function is grip strength [18]. Grip strength measurements further confirmed the results on low physical performance. Grip strength decreased in aged mice on HFD, which corresponded with the reduced running distance (Fig. 2D). Indirect calorimetry during the treadmill test revealed reduced oxygen consumption (VO2) in the aged HFD group compared to the aged chow group (Fig. 2E). Additionally, carbon dioxide production (VCO2) was reduced in both HFD groups in contrast to chow groups, regardless of age (Fig. 2F). Delta VO2, representing the change in oxygen consumption during the run, was lower in aged HFD mice compared to both age-matched controls and young HFD mice (Fig. 2G). The maximum respiratory tx ratio (max RER) was lower in both HFD groups, with aged chow-fed mice also showing a reduced RER compared to young chow-fed mice (Fig. 2H), indicating a substrate switch due to high fat intake and to a lower extend also due to age.

Changes in physical performance are often attributed to a loss of muscle strength or alterations in muscle morphology or metabolism [19]. To further assess the observed changes in physical performance, we conducted an analysis of skeletal muscle with a focus on the quadriceps. Both overall muscle mass and quadriceps mass decreased with age, but no significant changes were observed in response to HFD in both age groups (supplementary Fig. S3A-B). Muscle fiber size analysis showed no significant differences due to age or diet (supplementary Fig. S3C). Furthermore, biochemical analysis of the muscle sarcopenia marker did not show any differences (Fig. S4). Therefore, we conclude that the impairment in physical performance in aged mice on HFD was not due to remodeling of skeletal muscle. Furthermore, principal component analysis of proteomic data of quadriceps muscle did not reveal significant differences specific to the aged HFD group (data not shown) and, therefore, cannot explain the physical malperformance compared to the other groups.

### Heart weight increased due to lipid accumulation and cardiomyocyte hypertrophy in old HFD mice

**Fig. 3:**
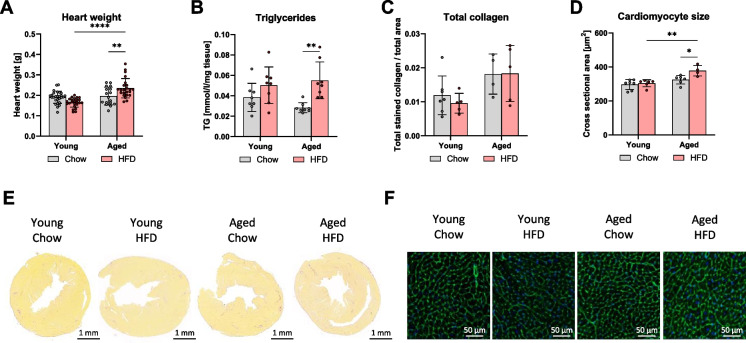
The increase in heart weight in aged mice on a high-fat diet was attributed to lipid accumulation and cardiomyocyte hypertrophy. Total heart weight (**A**). Triglyceride content per mg of tissue (**B**). Collagen content (**C**) with representative images (**E**), and cross-sectional area of cardiomyocytes (**D**) with representative images (**F**). Data are presented as mean ± SD. Statistical significance was tested using two-way ANOVA, with significance levels indicated as: *p < 0.05, **p < 0.01, ***p < 0.001, ****p < 0.0001

Physical performance is influenced not only by skeletal muscle but also by cardiovascular health [14]. Since, we could not detect any specific changes in the skeletal muscles of the aged HFD, we further investigated whether the decline in physical performance was related to changes in the heart. Consequently, we analyzed various cardiac parameters. While no significant changes in skeletal muscle and quadriceps mass were observed with HFD, heart weight increased in the aged HFD group compared to the aged chow group and the young HFD group. The young mice showed no changes in heart weight regardless of diet (Fig. 3A).

To explore the cause of this increase in heart weight, we performed various measurements. The total triglyceride content revealed a significant accumulation of cardiac triglycerides in the aged HFD group compared to the chow-fed controls, with only a tendency of HFD-associated changes in the young group not reaching the significance level (Fig. 3B). To assess potential morphological alterations of the heart, collagen staining was performed. However, no evidence of increased fibrosis by diet or age was found (Fig. 3C, E). Further examination of heart tissue through cardiomyocyte staining and measurement of cross-sectional area revealed cardiomyocyte hypertrophy, which was observed only in the aged HFD group in comparison to the age-matched chow group or the young groups (Fig. 3D, F).

### Aged mice showed diminished proteomic adaptability to HFD

**Fig. 4:**
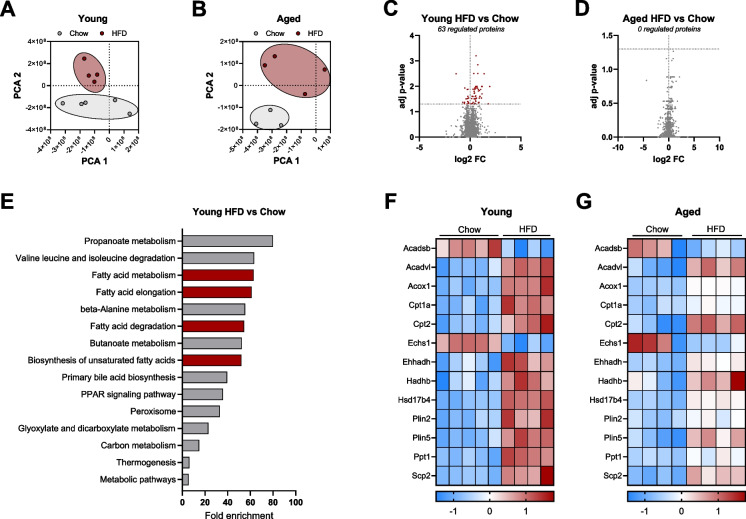
Aged mice on a high-fat diet exhibited reduced metabolic response. Principal component analysis (PCA) of all detected proteins comparing young chow vs. HFD (**A**) and old chow vs. HFD (**B**). Volcano plots showing the log2 fold changes of all detected proteins in young chow vs. HFD (*n* = 4–5; **C**) and old chow vs. HFD (*n* = 4; **D**). The left side of the plot corresponds to downregulated proteins, while the right side corresponds to upregulated proteins. Red data points indicate significantly regulated proteins (adjusted p-values, p < 0.05). KEGG pathway analysis of all significantly regulated proteins in young chow vs. HFD (**E**). Heatmap of selected significantly regulated proteins involved in lipid metabolism in young chow vs. HFD (**F**) and the same proteins in old chow vs. HFD (**G**), where blue indicates downregulation and red indicates upregulation

Since morphological analysis of the heart revealed differences specific for the aged HFD group, we conducted a proteomic analysis in heart tissue to gain an overview of protein patterns across the groups. Principal component analysis (PCA) plots revealed a clear separation between chow and HFD-fed mice in both age groups (Fig. 4A, B). Further analysis using volcano plots showed that young mice had 63 significantly regulated proteins when comparing chow to HFD (Fig. 4C). In contrast, no significantly regulated proteins were observed in the aged mice under the same conditions (Fig. 4D). To further investigate the 63 regulated proteins in young mice, we performed KEGG pathway enrichment analysis. Among the top 15 enriched pathways, four were related to fatty acid metabolism (Fig. 4E). Focusing on the proteins associated with fatty acid metabolism, we identified 13 regulated proteins. The heatmap for these 13 proteins displayed a distinct separation between chow and HFD in young mice (Fig. 4F). However, in the aged mice, the separation between chow and HFD was less pronounced, indicating diminished regulation of these fatty acid metabolism-related proteins in the aged cohort (Fig. 4G).

### Systolic heart function in aged mice was reduced due to HFD feeding

**Fig. 5:**
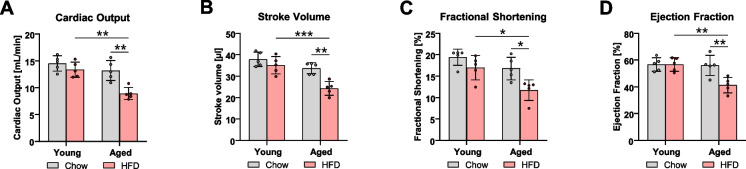
Aged mice developed a heart failure-like phenotype as a result of the high-fat feeding. Echocardiographic analysis of cardiac output (**A**), stroke volume (**B**), fractional shortening (**C**), and ejection fraction (**D**). Data are presented as mean ± SD. Statistical significance was tested using two-way ANOVA, with significance levels indicated as: *p < 0.05, **p < 0.01, ***p < 0.001, ****p < 0.0001

Given the morphological and metabolic changes observed in the hearts of aged HFD mice, we performed echocardiographic analysis to assess cardiac function. Aged HFD mice exhibited significant impairment in systolic function, measured as a reduction in cardiac output, stroke volume, and fractional shortening compared to chow-fed controls (Fig. 5A–C) (for the detailed echocardiographic parameters, see supplementary Fig. S5). Additionally, ejection fraction decreased to 41.4 ± 5.62%, indicating a heart failure phenotype with reduced ejection fraction (Fig. 5D).

## Discussion

Given the association of obesity with shortened life expectancy and an increased risk of cardiovascular diseases, this study aimed to investigate the interplay between obesity and age, focusing on physical performance and cardiac function. While previous research has predominantly examined either obesity or aging in isolation, the combined effects of these factors on skeletal muscle and cardiac metabolism and function remain underexplored. Our findings highlight the importance of age in modulating the response to HFD. We demonstrated that aged mice on HFD experienced physical functional impairments and could demonstrate an impairment of cardiac function. However, the role of the muscle in the development of such a physical malfunction can not be ruled out and needs to be investigated in a more detailed approach. The proteome response to HFD was impaired in the heart of aged HFD mice compared to their younger counterparts. Furthermore, lipid accumulation and cardiomyocyte hypertrophy in aged mice fed HFD contributed to a decline in systolic function, as measured by echocardiography. These results suggest that young mice possess metabolic alterations in the heart that protect against the detrimental effects of nutritional overload, while aged mice lose this response, leading to cardiac functional impairment and exercise intolerance.

Obesity is well-known to induce weight gain and adiposity in both humans and DIO mouse models [13, 20, 21]. Consistent with previous findings, our study demonstrated a significant increase in body weight and fat mass in both young and aged mice on HFD. However, our results also highlight the crucial role of age in modulating the response to HFD, with aged mice being more prone to rapid weight gain, primarily due to fat mass accumulation. While prior studies have similarly reported HFD-induced weight gain and fat accumulation, most have focused on a single age group [13, 20, 21]. Research on aging has consistently shown increased body weight and fat mass in aged mice compared to younger ones [22], which aligns with our results. However, the combined effects of HFD and age remain underexplored. Our findings are consistent with Jin et al. (2023), who also observed age-related differences in body weight and composition [23]. Interestingly, longer HFD exposure in other studies diminished the differences between young and old mice, as shown by Nunes-Souza et al. (2024) [24]. DIO mouse models remain a valuable tool for investigating metabolic changes associated with obesity [13, 21], though comparing studies can be challenging due to variations in intervention duration, diet composition, and animal age.

Previous HFD studies mainly focused on relatively young mice, with age ranging from 6 weeks [13, 20, 21] up to 3 to 6 months [23, 25, 26]. The age of “old” mice in the literature is typically between 22 to 24 months [22, 23, 27, 28]. However, we decided to use an earlier timepoint of 76 weeks (≈ 17.5 months). The aged group represents a stage in the mouse lifespan where the first substantial age-related physiological changes take place, but the age is not advanced enough for other aging-related conditions, such as cancer, inflammatory diseases, or type 2 diabetes, to significantly impact the analysis [29, 30].

Despite these challenges, our study aligns with existing literature regarding HFD-induced changes in body weight and composition.

Impaired physical performance is a well-documented consequence of obesity, often reflected in reductions in VO_2_max or peak VO_2_ during exercise [31–33]. In our study, no age-related effects were observed in the chow-fed groups, and the young HFD group did not show significant changes. However, aged mice on HFD exhibited a notable reduction in both running distance and VO_2_max. This is consistent with previous studies highlighting the negative impact of HFD on physical performance, mostly focusing on one age group. For instance, Marcaletti et al. (2011) found that young mice on a 16-week HFD showed reduced running distances and VO_2_max compared to controls [19]. The difference between their findings and ours may be explained by the longer HFD exposure in their study, which likely resulted in more pronounced performance declines, even in young mice. In contrast, Schefer and Talan (1996) reported no significant age-related differences in physical performance in adult and old mice without dietary interventions, which aligns with our findings regarding age effects [34]. However, other studies, such as Houtkooper et al. (2011), have reported declines in running distance, VO_2_, and RER due to aging. [22]. The differences in age ranges between studies may account for these discrepancies.

In humans, the impact of obesity on physical performance is also well-established. For example, Álvarez-Jiménez et al. (2022) demonstrated a clear reduction in VO_2_max in individuals with metabolic syndrome compared to healthy young adults [31], while Hulens et al. (2001) reported a significant decline in VO_2_max in obese versus lean women [32]. Villareal et al. (2011) further showed that weight loss through exercise and dietary interventions significantly improved VO_2_max in obese individuals over a 12-month period [33]. While obesity consistently leads to reduced physical performance in both mouse models and humans, our study highlights a distinct age-dependent effect, which corresponds with our findings on weight gain and fat mass accumulation.

Grip strength, a strong predictor of mortality risk and cardiovascular health [35], has been shown to decline with obesity [36]. In our study, grip strength decreased significantly in aged mice on HFD, but not in young mice. The decrease in muscle strength due to HFD has been reported previously [23]. Furthermore, in our study, we observed no effects of diet on muscle mass, quadriceps mass, or fiber size, though the mice showed an overall loss in muscle mass with age. This contrasts with findings by Jin et al. (2023), who reported reductions in quadriceps mass and fiber size with both aging and diet [23]. Other studies have similarly shown no changes in muscle mass with HFD in young mice [37], while some report increased muscle mass or fiber size with obesity [38, 39]. In summary, in our research, we did not observe significant changes in skeletal muscle that could explain the impaired physical performance, suggesting that other factors, such as cardiac function, may play a more prominent role.

Interestingly, the aged HFD group showed changes in cardiac morphology. Cardiac remodeling is a well-established process that occurs in response to both obesity and aging, and is characterized by structural changes in the heart [1, 8]. In the context of obesity and aging, the heart typically undergoes overall hypertrophy, with the left ventricle being particularly affected [40, 41]. On the cellular level, obesity causes a reduction in the number of cardiomyocytes, while at the same time inducing hypertrophy of the remaining cardiomyocytes. This remodeling disrupts normal heart function, as the hypertrophied cardiomyocytes struggle to maintain efficient contraction, ultimately leading to functional impairment and increased risk of heart failure [8, 42, 43]. Although the individual effects of obesity and aging on cardiac remodeling are well documented, the combined impact of both conditions is not as thoroughly understood. In our study, we observed significant cardiac morphological changes, but only in aged mice fed an HFD. The most notable finding was the increased heart weight in the aged HFD group compared to the young mice on HFD. This increase in heart weight is likely attributable to hypertrophy of the cardiomyocytes. Our findings are consistent with existing literature that demonstrates the detrimental effects of obesity or aging on the heart studies [42, 44, 45][8]. However, our study emphasizes the crucial role of age in modulating the cardiac response to nutritional overload.

Several studies have shown altered cardiac proteomes, particularly due to alterations in fatty acid (FA) metabolism in individuals with obesity or in aging [28, 46–49]. To further investigate alterations in the cardiac proteome, we utilized a proteomics approach. In our study, HFD feeding resulted in distinct alterations in the protein profiles of both age groups. Notably, there were no significantly regulated proteins in the aged mice with DIO compared to the young HFD mice, indicating a loss in proteome response to nutritional overload with age. This suggests that aged mice on HFD may experience metabolic dysfunction, potentially contributing to impaired cardiac function. While HFD induced an upregulation of FA metabolism proteins, aged mice on HFD did not show this response. It has been postulated that an early adaptive response to fat overload in mice is the upregulation of FA metabolism, particularly FA oxidation, in the heart, which can protect from lipid accumulation and cardiac dysfunction [50]. However, long-term HFD feeding can lead to an imbalance of FA uptake and oxidation, leading to lipid accumulation [51, 52]. Similar mechanisms have been observed in the context of aging [46, 53, 54]. This could explain the protective effect seen in the young mice on HFD, in which upregulation of FA metabolism proteins shown in the proteome helps to protect the heart from pathophysiological alterations after HFD exposure. To determine whether the observed changes in cardiac metabolism and morphology contributed to impaired heart function and the loss of physical performance, we assessed cardiac function. Our results revealed that heart function was significantly impaired in the aged HFD group compared to both the aged chow group and the young HFD group. The effect of HFD or Western diet on heart function was described earlier [55–57]. However, the effect in the content of aging was not addressed extensively before. Therefore, we chose a dietary intervention where young animals did not show extensive responses, to test whether aged animals did already show malperformance. This loss of function can be attributed to cardiomyocyte hypertrophy, lipid accumulation, and diminished cardiac metabolic response to HFD. The decline in heart function and the development of heart failure (HF) are well-established consequences of both obesity and aging [1, 8]. The development of HF is primarily driven by morphological and metabolic alterations that impair contractility and overall heart function [8, 58]. Moreover, HF is strongly associated with limitations in muscular function. Patients with HF often exhibit changes in skeletal muscle function, which, along with impaired heart function, lead to reduced exercise capacity [11]. This is further supported by studies that report decreased VO2 and shorter 6-min walking distances in patients with both HFrEF and HFpEF [59]. Similarly, a rat model of HF demonstrated reduced running distance following HF induction [60]. These findings align with our results, where the aged HFD group, being the only group to develop HF, also exhibited a marked decline in physical performance. These results underscore the critical interplay between skeletal muscle function and cardiac performance, particularly in the context of HF.

In conclusion, recent findings suggest that obesity-induced metabolic changes impair heart function, leading to reduced physical performance. Our results confirm that DIO leads to metabolic alterations and cardiac dysfunction, but these effects are strongly age-dependent. Based on the data presented here, we propose the hypothesis that the limited response of fatty acid metabolism to HFD is one of the driving forces leading to cardiac malfunction in aging hearts.

## Limitations

While this study provides valuable insights, future research should include additional age groups to better define the timeline of these effects. We intentionally chose not to use very old mice (24 months or older) as it is well-documented that older organisms have largely lost their capacity for adaptation and often present with other age-related comorbidities, such as cancer or inflammatory diseases. Instead, it is more prudent to use “middle-aged” animals, which represent a stage in the mouse lifespan where age-related physiological changes have begun but do not yet result in a diseased phenotype [29, 30]. Furthermore, human studies have shown that the peak prevalence of obesity is observed in middle-aged individuals (50 to 60 years of age) [2, 61], further supporting the relevance of our chosen age group. Follow-up experiments are required to comprehensively understand the mechanisms underlying the loss of metabolic response, with a particular focus on FA metabolism. Proteomic data provided initial evidence of maladaptive responses in FA metabolism in aged mice on HFD. However, more detailed analyses of the associated pathways and processes are necessary to elucidate the precise mechanisms responsible for these alterations. Such investigations will be crucial in providing a clearer understanding of the metabolic dysfunction observed in this context.

We restricted our studies to male animals in order to restrict the number of animals used to a minimum. Several studies have demonstrated that female mice are more resistant to HFD-induced metabolic dysfunction and cardiac remodeling compared to males. These sex-based differences are often attributed to the actions of estrogen on mitochondrial function, lipid metabolism, and oxidative stress pathways. We are aware that metabolic heart failures are also formed in woman. However, there are multiple conditions to observe using female mice, so that we restricted our initial experiments to males. After revealing that heart conditions are a driving force of age-related changes, it is prudent to use in further studies both sexes to investigate in detail the mechanisms underlying these changes.
